# Expansion Injection Molding Process Using Clamping Force for Melt Compression

**DOI:** 10.3390/polym16030424

**Published:** 2024-02-02

**Authors:** Joon Hyoung Park, Sun Kyoung Kim

**Affiliations:** Department of Mechanical System Design Engineering, Seoul National University of Science and Technology, Seoul 01811, Republic of Korea

**Keywords:** injection molding, compression, expansion, MoldFlow

## Abstract

Melt expansion followed by compression has been utilized for high-speed filling. In general, this technology was developed for a machine level. Recently, mold-level technology has been tried. In this study, an expansion injection molding process was examined, which included compressing a polymer melt through cylinder action facilitated by the movement of the platen, followed by the expansion of the polymer melt into a mold cavity. A mold system including temperature control and valve actions, similar to hot runner systems, was designed and built. The test results show good filling when the injection pressure was high. Simulations were also carried out, highlighting consistent pressure and filling trends, while revealing limitations tied to the characteristics of the state model. This research indicates promise for expansion injection molding through platen compression but emphasizes the need for the seamless integration of valve action with the injection molding machine for large-scale production.

## 1. Introduction

The injection molding process, using thermoplastic polymer pellets derived from the extrusion process, has firmly established itself over several decades as the fundamental method for manufacturing mass-produced plastic components [1]. Nevertheless, conventional injection processes possess inherent limitations [2]. In particular, difficulties arise in creating excessively thick parts due to dimensional shrinkage or because of constraints on thickness due to high viscosity, thereby restricting the attainable thickness [3]. On the other hand, producing thin components through injection molding requires overcoming challenges such as filling issues associated with the high viscosity of the molten melt and coping with difficulties related to moldability.

Reducing product thickness is crucial for achieving the desired geometric shape and provides production advantages by minimizing cooling time during the injection molding process [4]. Pursuing thinner thicknesses is especially beneficial for productivity, enabling the production of precise components with shorter cooling times [5]. Typically, the required cooling time is directly proportional to the square of the thickness [6]. As a result, numerous ongoing research endeavors are dedicated to the refinement of micro-injection molding techniques and the advancement of thin-product manufacturing [7,8]. For micro-injection [9,10,11,12] and thin-walled medical components [13], researchers have investigated replication characteristics during filling.

As thickness decreases, the proportion of the skin layer to the overall thickness increases [14]. In these cases, prolonged solidification time can significantly impact crystal growth, creating challenges in quality control for injection-molded products [15]. Rapid filling is especially advantageous for thin-skin thicknesses, posing difficulties in shaping thin-walled parts, including micro-injection molding, where addressing hesitation in macro-cavities proves challenging [16,17]. This approach provides benefits like suppressing viscous heating, enhancing melt homogeneity, and potentially reducing peak pressure during filling.

To overcome the challenge of filling thin sections, achieving rapid filling through increased injection speed becomes a valuable technique [18,19]. For this purpose, injection molding machines with hydraulic reservoirs have been employed [20]. However, the associated costs and maintenance requirements make such machines less popular for general use. An alternative method to achieve high-speed filling characteristics is known, involving the use of a conventional injection molding machine and utilizing mold technology during the initial phase, eliminating the need for high-speed injection machines. During the use of high-speed injection machines, resin overfilling has been observed, attributed to resin compression.

The phenomenon should be prevented through control, but on the other hand [21], the expansion characteristics can also be utilized in injection molding [22]. Additionally, resin compression caused by the pin action of the hot runner has also been identified. Building on these observations, Vetter et al. proposed a technique involving modifying hot runner nozzles to implement an expansion injection molding (EIM) process [23]. In their study, they controlled the valve pin’s position to compress the resin using the pin’s movement before opening, subsequently allowing the resin to expand toward the gate upon pin opening. This approach employs a structure similar to the conventional hot runner, potentially leading to insufficient injection volume issues.

In this work, a dedicated research device is designed, implemented, and tested to assess the viability of augmenting injection volume with a conventional injection molding machine. Compressibility improvement will be achieved by constructing a device that gathers and compresses the melt, with a temperature-controlled cylinder being employed for this purpose. Subsequently, the cylinder will be connected to a slender disc-shaped cavity to evaluate its capability for expansion-induced filling. Ultimately, a systematic comparison of the results with numerical simulations will be carried out. The ensuing detailed information is provided below.

## 2. Methods

### 2.1. Process and Mold Design

To enhance the performance of the earlier expansion injection mold, which altered the existing hot runner nozzle [23], a new molding system was created. The primary objective of this development is to augment both the fill volume and reservoir pressure. Achieving this goal necessitates the incorporation of a piston with a larger cross-sectional area. In contrast to the previous method where the shutoff pin served as a compression piston, the new system employs a dedicated piston for this purpose.

**Figure 1:**
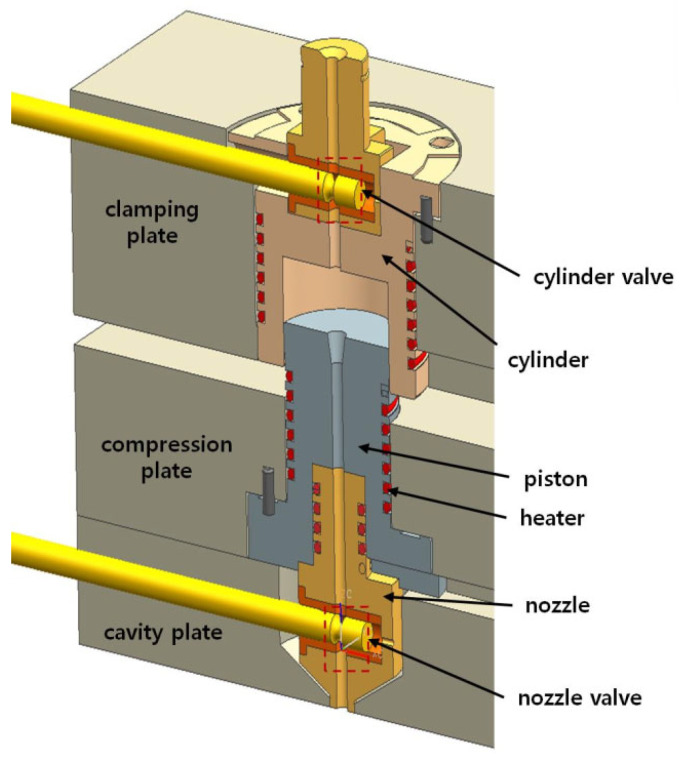
Structure of the mold.

In the proposed method, thermoplastic melt injected into a cylinder is subject to compression by movement of a platen. This concept is similar to pressurization in injection compression molding [24]. Note that pressure plays many useful roles in injection molding [25]. Then, the melt is expected to expand on entering the mold cavity. To realize this method, an experimental mold system is designed. Figure 1 shows a virtually assembled view of the core components constituting the cylinder.

There are three plates, which are the clamping, compression, and cavity plates. The clamping plate retains the cylinder that is a reservoir for the compressed melt. The piston, which holds the nozzle, is assembled in the compression plate. The motion of compression plate relative to the clamping plate changes the volume inside the cylinder. Two pin valves are employed to control the inflow, the compression, and the outflow. The pins have a neck near the end to allow melt flow when the neck is aligned with the runner. Moreover, to control the melt temperature, wire heaters are embedded in the cylinder, piston, and nozzle. A load cell is connected to the cylinder cavity using a rod parallel to the plates. The cylinder and nozzle valves, which are pneumatically operated, are placed between the plates. Because the piston assembly is retained inside, the distance between plates controls the volume.

**Figure 2:**
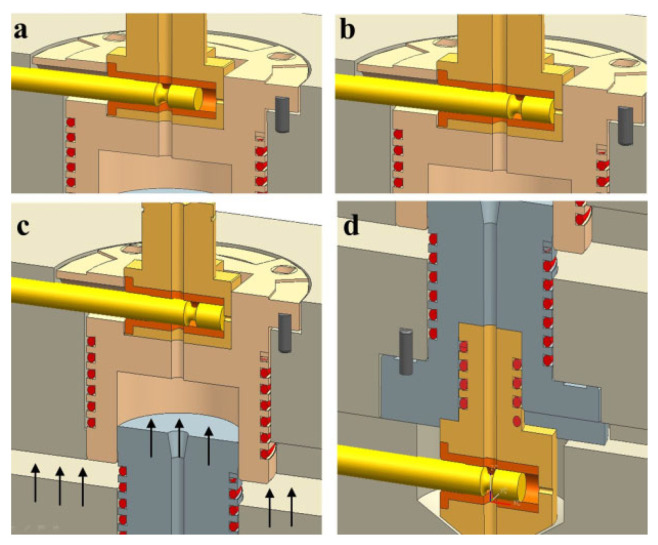
Sequence of the movement of valve pins, platen, and cylinders: (**a**) Aligning the upper pin to open the cylinder inlet; (**b**) Aligning the upper pin to close the cylinder; (**c**) Initiating piston upward movement for compression; (**d**) Aligning the lower pin to open the cylinder outlet.

Figure 2a,b shows the moment when the cylinder valve is closed and opened, respectively. Figure 2c shows cylinder cavity surrounded by the cylinder body and the piston. Figure 2d shows the nozzle valve at the open position. The processing procedures are as follows: Initially, the cylinder valve is opened to allow melt flow into the cylinder with the nozzle valve closed. After the measured amount of melt is injected into the cylinder through the cylinder valve, it should be closed as in Figure 2b. Then, the piston should move toward the cylinder top to compress the melt inside as shown in Figure 2c. On completion of compression, the nozzle valve is moved to the open position as shown in Figure 2d to inject the compressed melt into the mold cavity by expansion. When the pressure inside the cylinder becomes significantly lowered and the expansion is considered complete, the cylinder valve is opened to induce additional filling or to apply compaction pressure. This final state is shown in Figure 1.

It is quite difficult to determine the diameter of the piston. Given a volume of the uncompressed volume of the cylinder cavity, diameter should be bigger to make the piston stroke short. In this setup, a long stroke necessitates perfect alignment and machining of mold parts because clamping motion is utilized. Three major difficulties arise when the cylinder diameter is increased. First, the force for melt compression becomes larger proportional to the square of the diameter. Second, the tolerance between the cylinder and the piston is extremely difficult to control. Third, it is very difficult to install other parts including melt pressure sensor, heater, and valves. Considering all these, the diameter was chosen as 60 mm. In addition, a heating wire was wound on the piston to control the temperature.

### 2.2. Tooling

Most of the parts of the mold in Figure 1 can be built using standard parts except the cylinder and the piston. It is very difficult to control the tolerances between the piston and the cylinder. To allow assembly and movement of the piston inside the cylinder, there should be significant clearance. However, because the cylinder cavity is supposed to be under high pressure and high temperature, such a clearance will cause huge leaks. In addition, there are thermal expansions of cylinder and piston that are quite complicated to predict. Thus, it is concluded that prevention of leakage by the tolerance control is impracticable.

**Figure 3:**
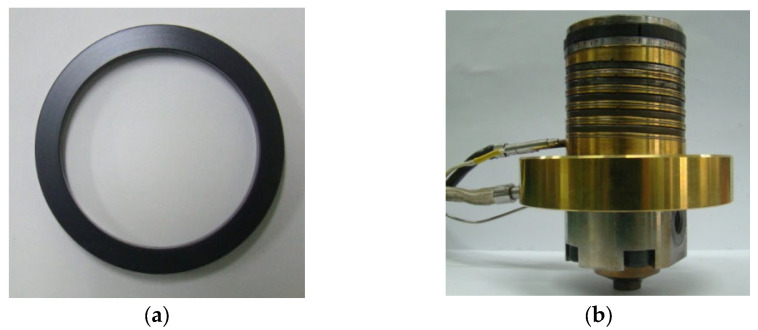

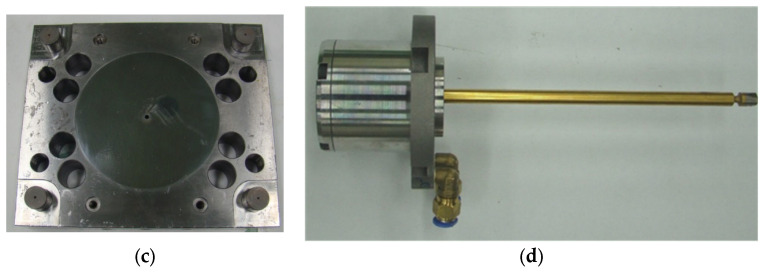
Fabricated mold parts: (**a**) ring; (**b**) piston; (**c**) cavity plate; (**d**) valve gate pin.

**Figure 4:**
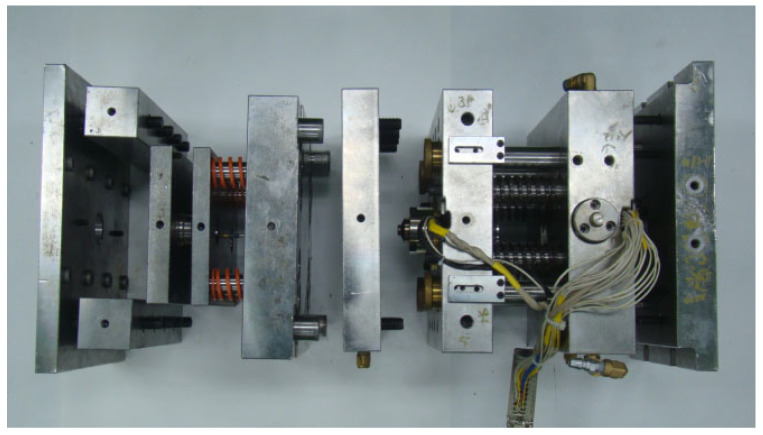
The exploded view of the manufactured mold.

In this work, a polymer ring is designed and installed for sealing the melt. The ring is fabricated as shown in Figure 3a by machining PBI (polybenzimidazole) rod (Celazole, U-60) of which HDT (heat distortion temperature) is 435 °C. Because the linear thermal expansion coefficient of PBI (33 μm/mK above 500 K) is much larger than that of the cylinder, a secure seal is ensured between the ring and the inner cylinder wall at the operating range. It is known for high-temperature applications [26]. Figure 3b shows the assembled piston with heating wire and a thermocouple. Figure 3c shows the cavity plate with an ejection pin hole in the center. Moreover, the valve pins in Figure 1 are fabricated as shown in Figure 3d. Figure 4 shows an exploded view of the built mold. Refer to Appendix A for further details on tooling.

### 2.3. Instrumentation

**Figure A3:**
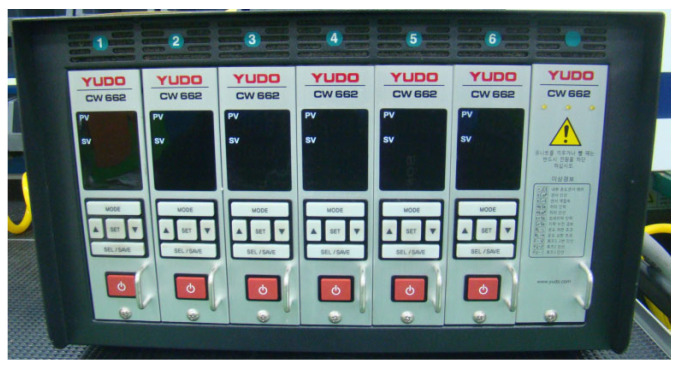
Hot runner controller employed in this work for control of the cylinder temperature (CW662, Yudo, Hwaseong, Republic of Korea).

The entire heating area is divided into 6 separate zones where the temperature of each zone is controlled by a separate controller. All zones utilize identical controllers, and the wire bundle in Figure 4 is connected to the controller array. The controller is a conventional one, which is usually used for injection molds with hot runners (Yudo, Hwaseong, Republic of Korea, CW 662). To each controller, a K-type thermocouple is wired from each zone. Moreover, an electric injection molding machine (Woojin Plaimm, Boeun, Republic of Korea, TE110, clamping force of 1079 kN, Figure A3) is employed to test the developed mold. To measure the melt pressure inside the cylinder cavity, a load cell (CAS, Yangju, Republic of Korea, LSC-2, up to 19.6 kN, strain gauge type) is employed. The pressure inside the cylinder cavity is relayed to the load cell via a cylindrical rod 8 mm diameter because its direct embedding in the cylinder wall is not allowed due to high temperature. Thus, the pressure is calculated as the measured force divided by the cross-sectional area of the rod. The purpose of the measurement is to obtain the initial pressure of the cylinder prior to opening the nozzle valve.

### 2.4. Materials and Conditions

Because this study is focused on development of a molding technology, a thermally stable general material is chosen. This work presents the result with a HDPE (high-density polyethylene, Prime Polymer, Tokyo, Japan, Hi-Zex 2100JH), which is an injection-molding-grade resin. The melting temperature is 129 °C, and the melt flow index is 9.1 g/10 min, with a reference density of 951 kg/m^3^.

The melt temperature is set as 230 °C for all the cases in this work, and the mold temperature is controlled at 60 °C. The disks are molded by EIM with several different initial pressures in the cylinder. To compare the EIM with the conventional IM (injection molding), molding experiment by IM at the maximum possible injection velocity, which is 400 mm/s, is also conducted. At 230 °C, the melt can be compressed approximately 13% under 200 MPa based on the pvT data.

### 2.5. Equations

There are a couple of important points in this simulation. On opening of the nozzle valve, filling is initiated. This should be performed after the velocity pressure switchover. Before the switchover, this pressure-driven flow cannot be simulated. In the postfilling (packing) phase, compressibility can be taken into account and the melt is treated as the slightly compressible fluid [27]. In addition, because the initial velocity is extremely high, the numerical mesh should be fine enough.

To have the cylinder uniformly pressurized, it needs a significantly long time. However, we found that it was not possible in MoldFlow 2016 to keep the cylinder uniformly pressurized with both the valves closed. Thus, the maximum injection pressure was set as the measured pressure to mimic the experimental condition. This means the cylinder is pressurized at the injection pressure and then the valve action takes place.

The viscosity in MoldFlow is described as a function of temperature *T* and shear rate $\dot{\gamma }$ by the Cross-WLF model during calculation.

(1) $$\eta (\dot{\gamma },T)=\frac{\eta_{0}(T)}{1+(\eta_{0}(T)\cdot \dot{\gamma }/\tau {)}^{1-n}}$$

where $\eta_{0}=D_{1}\exp\left[-A_{1}(T-D_{2}-D_{3}p)/(A_{2}+T-D_{2})\right]$ for $T\geq D_{2}$ and $\eta_{0}=\infty$ for $T<D_{2}$. Here, for the selected material, the coefficients are *D*_1_ = 4.4 $\;\times 10^{15}\mathrm{Pa}\cdot s$, *D*_2_ = 153.15 K, $D_{3}=1.2\times 10^{-7}$ K/Pa, *A*_1_ = 34.339, *A*_2_ = 51.6 K, *n* = 0.3542, and *τ* = 55,200 Pa [28]. Note that the viscosity can be increased due to $D_{3}$. Especially for high-speed flow, the effect is considerable [19].

The pressure-dependent density is modeled by the Tait equation, where the specific volume is of the form

(2) $$v(T,p)=v_{o}(T)\left[1-C\ln(1+\frac{p}{B(T)})\right]+v_{g}(T,p)$$

where $v_{o}(T)=b_{1}+b_{2}\bar{T}$, ${B\left(T\right)=b}_{3}\exp\left[-b_{4}\bar{T}\right]$, $v_{g}(T,p)=b_{7}\exp\left(b_{8}\bar{T}-b_{9}p\right)$, and $\bar{T}=T-b_{5}$. The constants in Equation (2) for the HDPE are presented in Table 1.

Based on Equation (2), the fillable volume by expansion under isothermal conditions can be estimated by

(3) $$\Delta V=\frac{V_{0}}{v_{0}}\left[v_{F}-v_{0}\right]$$

where $V_{0}$, $v_{0}$, and $v_{F}$ are the volume of the cylinder at the compressed state, the initial specific volume, and the final specific volume, respectively. Note that the specific volumes are calculated by Equation (2).

**Figure 5:**
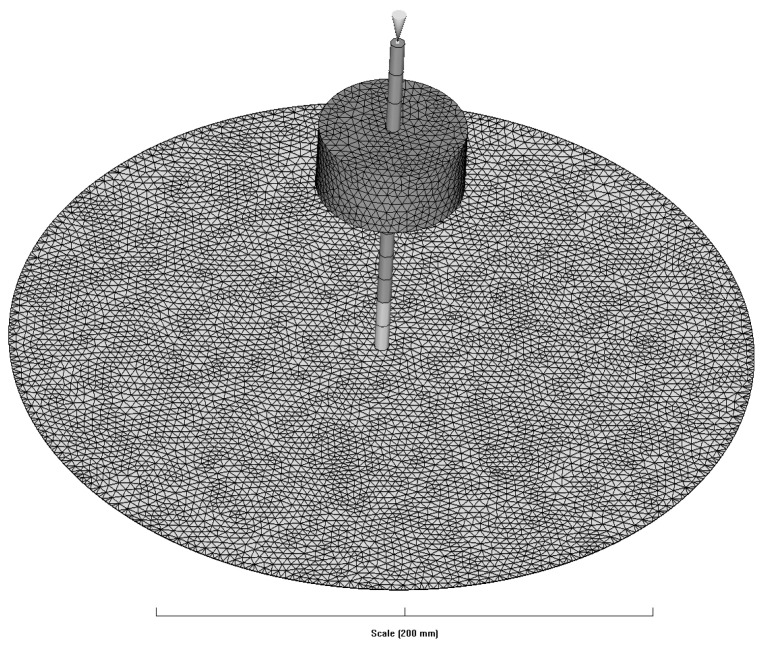
The geometry and mesh for the simulation in MoldFlow.

Figure 5 shows the geometry and mesh of the simulation. The cylinder was modeled based on the dimensions when compression was completed. To simulate various cases with different initial pressures, the diameter of the cavity is set as 300 mm. A total of 247,850 tetrahedral elements are used. At the top position of the hot runner, the inlet gate is located. For runners, beam meshes are employed. The upper part is treated as a hot runner, while the lower part is treated as a cold runner.

### 2.6. Molding System

As is evident from the design of this EIM system, the cylinder pressure is limited by the clamping force. The melt pressure could not be increased over approximately 130 MPa with the selected injection molding system. To increase the melt pressure, the clamping of the injection molding machine should be increased. This is possible by replacing the injection molding machine with a bigger one. Otherwise, the cylinder diameter needs to be reduced. To maintain the same cylinder volume even with a reduced diameter, stroke of the piston has to be increased considering that the clamping force equals the pressure multiplied by the cross-sectional area. The piston ring made of PBI can prevent possible melt leak while the melt temperature is maintained up to 270 °C. Both the valves are operated by action of the pneumatic cylinders. Injection of the melt in the cylinder to the mold cavity can be conducted in accordance with operation of the injection molding machine.

### 2.7. Test Cases and Conditions

In this study, experiments compare conventional injection molding (IM) and EIM. The IM case is denoted as IM, with the injection speed set at 400 mm/s, representing the maximum achievable speed for the utilized injection molding machine. In the case of EIM instances, we intended to conduct experiments by filling the interior of the cylinder at an injection speed of 100 mm/s and then expanding it by opening the valve during actual injection. Because this study aims to observe only the expansion characteristics, the connection with the injection machine is blocked, and the state depicted in Figure 2b is maintained without any packing.

When employing EIM, tests were conducted across five distinct scenarios, corresponding to internal cylinder pressures of 29, 39, 59.5, 78, and 117 MPa, labeled as EIM1, EIM2, EIM3, EIM4, and EIM5, respectively. Because the focus is on analyzing filling characteristics, the experiments were conducted without the use of a holding pressure process. Additionally, each case underwent ten repetitions of the tests.

## 3. Results

### 3.1. Diameter

**Figure 6:**
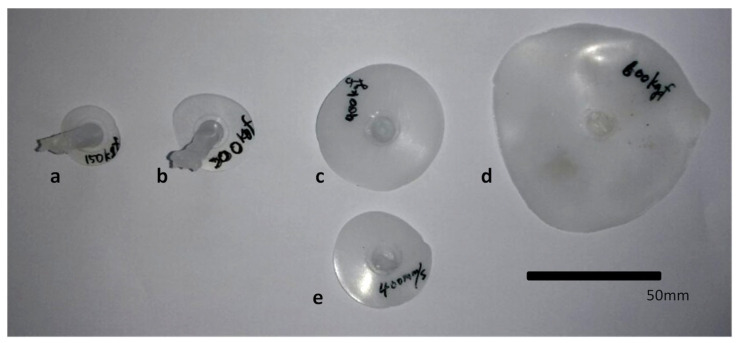
Molded articles alongside compression conditions: (**a**) EIM1; (**b**) EIM2; (**c**) EIM4; (**d**) EIM5; (**e**) IM.

Figure 6 shows the disks molded by the EIM and the conventional IM. The shape of the molded disk is quite irregular at a higher melt pressure. For each sample, the radii of the disks were measured using a caliper in 12 different directions rotating 30° at a time. The measured values were taken as the diameter of a circle, which has the same area as the closed curve interpolating these measurement points.

Based on a visual comparison, the last two EIM cases in the figure resulted in molded disks larger than that by the conventional IM at 400 mm/s, which was the maximum injection speed of the machine. It can be concluded that the EIM can fill the cavity that cannot be filled by the IM as in the EIM4 and EIM5 cases. From the observed injection volume of EIM5 in this figure, it is anticipated that utilizing EIM for injecting micro- or thin medical parts is entirely feasible.

**Figure 7:**
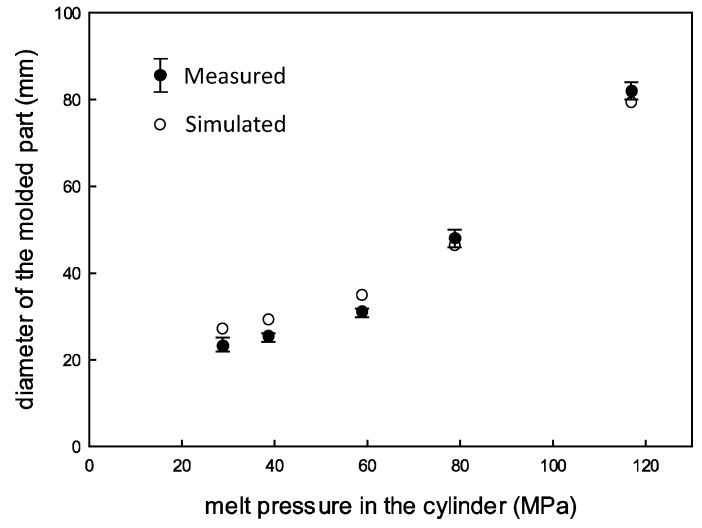
Measured radius distribution along with the initial melt pressure for all EIM cases (from EIM1 to EIM5).

Figure 7 shows the measured and simulated diameters of the molded disks along with the initial melt pressure in the cylinder for all EIM cases. The diameter increases along with the pressure, and the data are more scattered for cases with higher cylinder pressure. The predicted diameter, estimated using Equation (3), is more than twice as large as those diameters shown in the figure. For instance, the diameters are 72.9 mm for EIM1 and 186.6 mm for EIM5. This disparity is attributed to the isothermal assumption in Equation (3). The reduction in melt temperature within the cavity, caused by the low mold temperature, heightens shear friction in the mold, leading to an expansion halt at a pressure that balances with the friction. To mitigate this effect, a faster filling process is recommended. Consequently, the ratio of the predicted to measured diameters is lowest for EIM5 and highest for EIM1.

**Figure 8:**
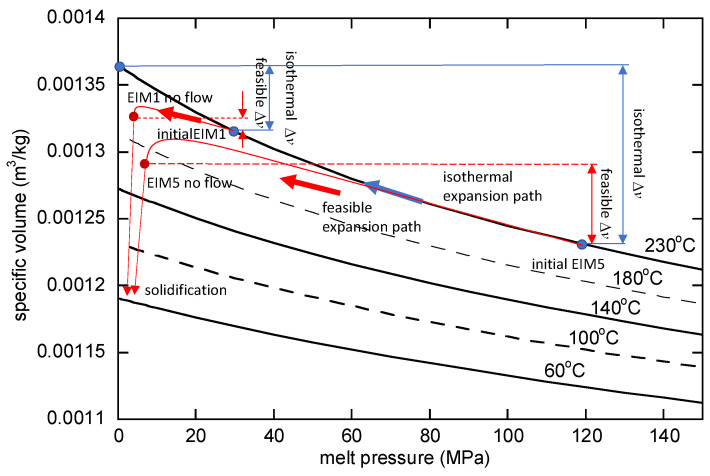
Feasible and isothermal expansion paths for EIM1 and EIM5.

Figure 8 illustrates the v-p curve according to temperature. In the case of EIM5, expansion occurs from the initially compressed state at 117 MPa and a temperature of 230 °C, which is the initial melt temperature. If it were an isothermal condition, expansion would follow the 230 °C line on the graph. However, within the cavity, cooling occurs, causing expansion toward a lower specific volume than indicated by the 230 °C line. The feasible expansion paths for EIM1 and EIM5 shown in Figure 8 are an approximate representation obtained by estimating the specific volume from the measured values and approximating the pressure that roughly balances with shear stress from the mold temperature. It is evident that the expansion effect decreases as the temperature decreases. Therefore, to achieve the EIM effect, it is deemed necessary to increase the initial pressure, thereby minimizing temperature reduction by increasing the injection speed. In the case of EIM5, the melt flow continues for a prolonged duration, leading to a final temperature lower than that of EIM1. Consequently, the flow comes to a stop at a higher pressure.

**Figure 9:**
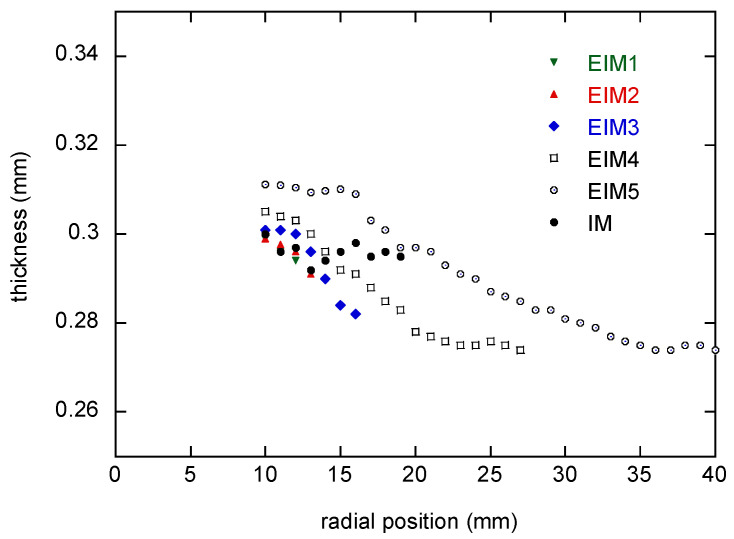
Radial thickness distribution of the molded article for EIM4, EIM5, and IM.

Initially, the flow from the top gate impinges onto the bottom cavity wall and then flows radially through the parallel cavity with a uniform thickness of 0.3 mm. The flow is supposed to stop under the strong pressure gradient resulting in a short shot. Because the melt is solidified without pressurization for packing, the gradient in the filling phase should affect the thickness. The thicknesses were measured with a micrometer along the four different radial lines at every 1 mm. Figure 9 shows the thickness averaged for all ten samples.

The thicknesses of the EIM cases decrease monotonically along with the radial position. On the other hand, the IM case shows quite a uniform thickness. This means it is important to set the initial cylinder pressure properly to mold a part with dimensional stability.

### 3.2. Simulation

**Figure 10:**
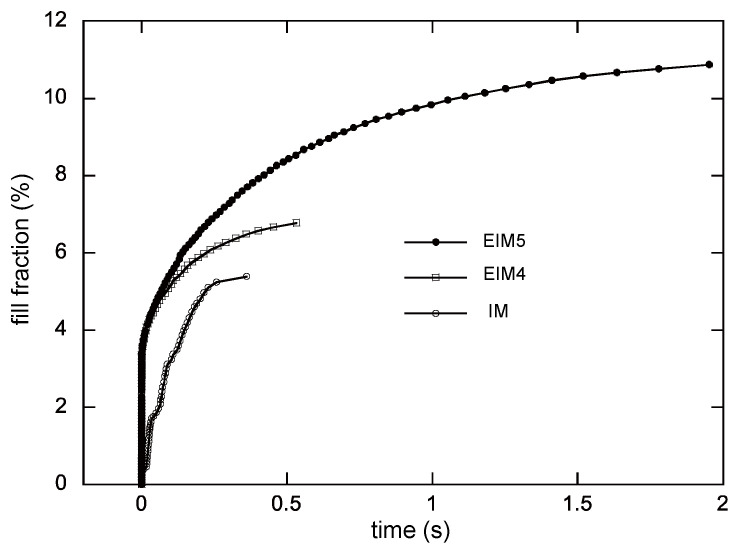
The simulated fill fraction along with time.

**Figure 11:**
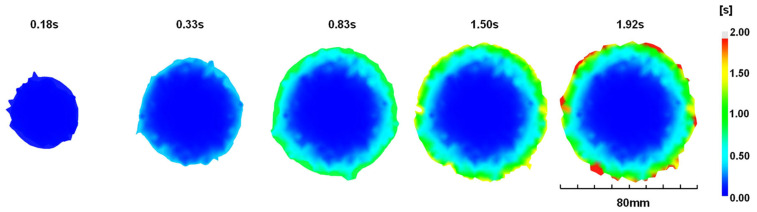
Simulated flow front for EIM5.

According to the aforementioned method, the three different experimental cases in the previous section were simulated. Figure 10 shows the simulated fill fraction versus the fill time for all the cases for the experiments. Instantaneous increases in the fill faction are observed in the EIM cases. After that initial phase, the fill fraction grows slowly and then eventually reaches the final fill fraction. In the normal injection by the screw, there is no such fast filling. Note that the time required for filling the cylinder is excluded in the presented figures by setting the time when the nozzle valve is opened as 0 s. Figure 11 shows the filled disks along with the fill time. Again, the result shows fast filling. Refer to Figure 7 again for the simulated values of the final diameters. The simulated values for EIM1, EIM2, and EIM3 surpass the measured values slightly, while EIM4 and EIM5 exhibit slightly smaller values. On the whole, there is good agreement between the simulation and the measurements.

**Figure 12:**
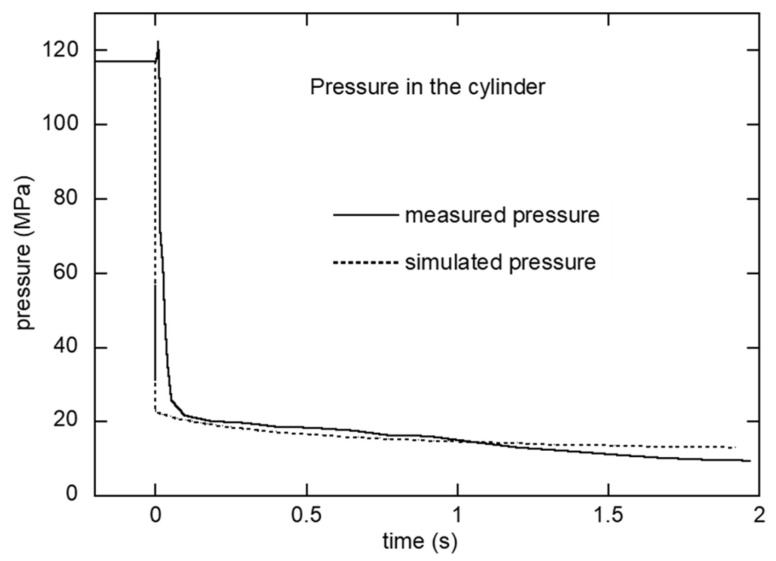
Measured and simulated pressure in the cylinder over time for EIM5.

Figure 12 compares the simulated and measured pressures inside the cylinder over time. As the load cell measures the pressure inside the cylinder, the simulated pressure was probed at the center of the cylinder. The simulated pressure dropped immediately from its initial value to 22 MPa within 1 ms, while the measured pressure took approximately 0.1 s to drop. As a result, a discrepancy between the simulated and measured pressures was observed. The abrupt drop in simulated pressure is likely attributable to the instantaneous response of the state equation, which lacks a time-dependent component. Meanwhile, the load cell employed in this study lacks the requisite speed to accurately capture this rapid pressure decline. Consequently, it is anticipated that the actual pressure curve will fall between these two curves. A rate-dependent model would alleviate this discrepancy [29].

**Figure 13:**
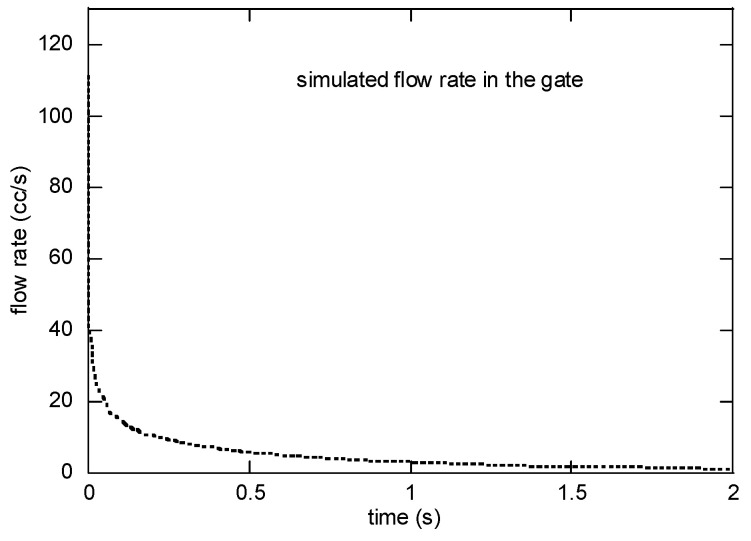
Simulated flow rate at the gate for EIM5.

The simulated flow rate and the radial velocity at the gate are presented in Figure 13. The flow rate was initially up to 35 cc/s at 0.01 s and was reduced to 2 cc/s in 0.1 s. The velocity was 1.11 m/s right after the valve opening. On a personal computer with Intel i7 4775 K CPU, it took 1355 s for a single case.

### 3.3. Discussions

As mentioned in the introduction, the potential of EIM was implied by altering the hot runner nozzle structure in prior research. The cavity volume in that study was considerably small, being three orders of magnitude smaller, posing challenges in assessing feasibility for larger-scale injection applications. This study facilitated an estimation of the actual achievable injection volume through EIM by considering a larger cavity.

The finding confirmed that EIM exhibits rapid filling under high pressure, as illustrated in Figure 8. The significant thickness variation in EIM, evident from the results, suggests that relying solely on this factor would be insufficient to complete the process. Nevertheless, the necessity of achieving rapid initial filling and subsequent injection and holding processes, driven by additional screw movements, is acknowledged. Given the specified objective to verify the effects and potential of EIM, this particular aspect was not addressed. Furthermore, the simulation revealed a discrepancy at the initial time. This is considered primarily due to the estimation of the specific volume using a state equation.

In practical production processes, there is an anticipation of the need for the integration of the injection machine and EIM valves. This integration is deemed essential for seamless operations, underscoring a critical consideration for the broader application of EIM in real-world manufacturing processes. Moreover, the experimental mold structure proposed in this study is significantly large to the extent that it poses challenges in its application to mass production molds. To make it applicable, simplification and reduction are necessary, and there is a need to find cost-effective materials for sealing as well.

## 4. Conclusions

In this study, the process involving the compression of a cylinder serving as an accumulator and the subsequent expansion of the compressed polymer melt into the cavity through the movement of the platen in the injection mold was tested. As the cylinder needed to maintain the melt’s temperature, temperature control, similar to that in a hot runner system, was deemed necessary. To achieve this, existing hot runner valves and controllers were utilized. Given the cylinder’s diameter size issue, sealing was achieved using PBI (polybenzimidazole), a polymer with excellent high-temperature properties.

Using this mold, injection molding was performed to evaluate the filling characteristics achieved in the EIM process. The results show a significant level of filling when the injection pressure was sufficiently high. Additionally, simulations using mold flow analysis demonstrated similar characteristics. However, due to the nature of the pvT model, it exhibited a limitation where the initial expansion was simulated to occur excessively faster than in reality. Through this research, the potential of EIM utilizing the movement of the platen was explored, and it is expected that it could be applied to mass production injection processes if seamless integration with the valve structure of the injection machine can be achieved. It has been confirmed that a sufficient injection volume can be secured to apply this injection method to micro-injection or the injection of thin-walled parts.

To effectively apply this injection method, it is considered necessary to implement an automated system that integrates the control of measured pressure and temperature with the operation of the injection machine. Furthermore, to ensure the effective application of this method, it is judged that increasing the mold temperature is necessary, and for this purpose, research combining EIM and heat and cool is also deemed necessary.

## Figures and Tables

**Table 1:** Constants of the *pvT* equation [28].

*C*	0.0894
*b*_5_ (K)	414.5
*b*_6_ (K/Pa)	1.543 × 10^−7^
*b*_1*m*_ (m^3^/kg)	0.001274
*b*_2*m*_ (m^3^/kgK)	1.026 × 10^−6^
*b*_3*m*_ (Pa)	9.263 × 10^7^
*b*_4*m*_ (1/K)	0.004941
*b*_1*s*_ (m^3^/kg)	0.001075
*b*_2*s*_ (m^3^/kgK)	2.077 × 10^−7^
*b*_3*s*_ (Pa)	3.324 × 10^8^
*b*_4*s*_ (1/K)	2.46 × 10^−6^
*b*_7_ (m^3^/kg)	0.0001872
*b*_8_ (1/K)	0.05158
*b*_9_ (1/Pa)	1.023 × 10^−8^

Note the subscripts *m* and *s* denote the melt and solid phase, respectively.

**Figure A1:**
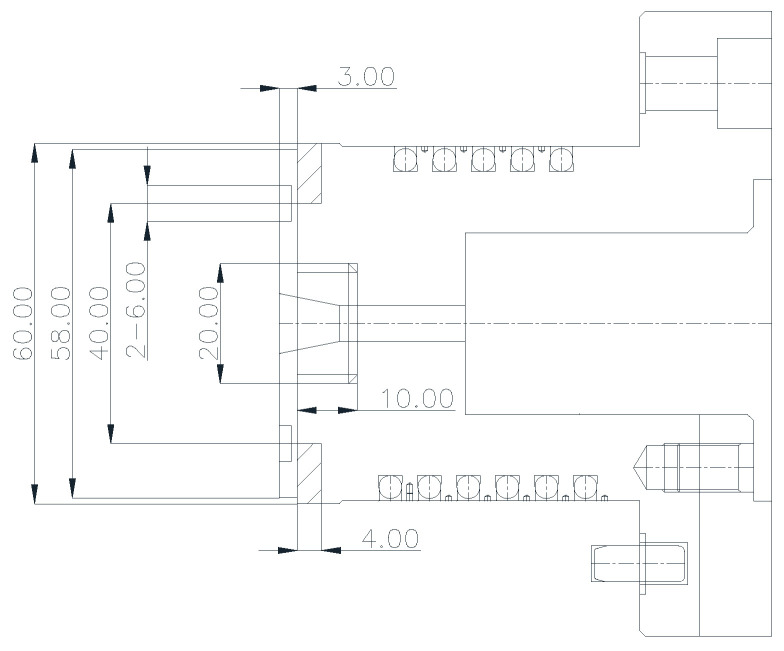
Design of the piston with heating coil. A heating wire is wound to control the temperature. All dimensions are in mm.

**Figure A2:**
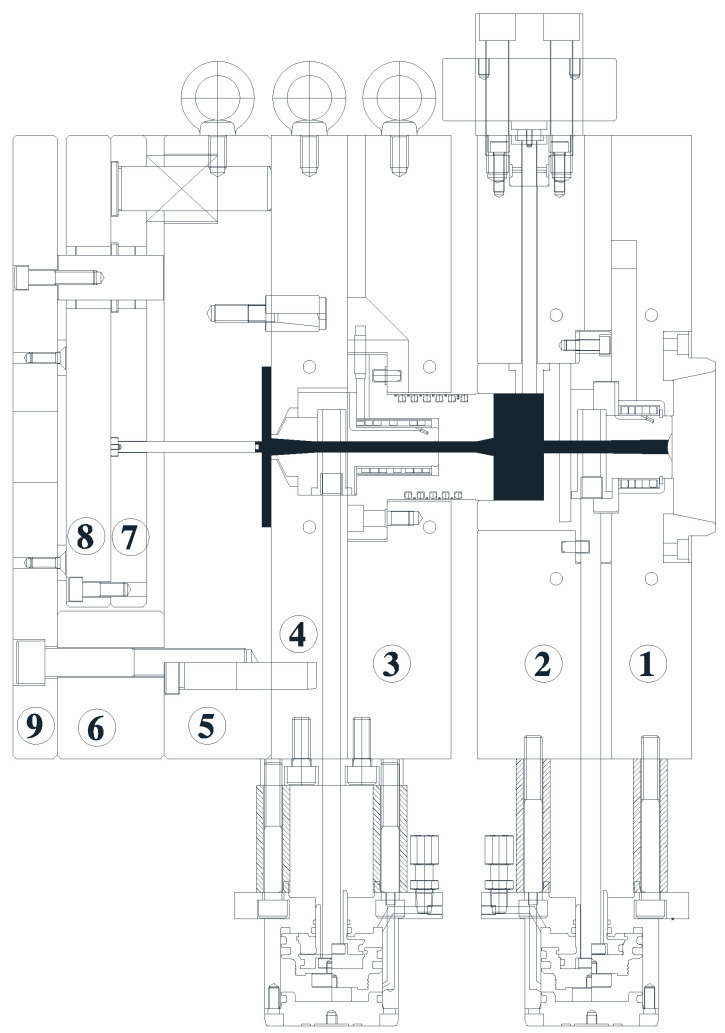
Assembled mold system with hydraulic valve drivers. The injection nozzle is connected to plate 1. The cylinder cavity is formed by plate 2 and the piston. A load cell is connected to the cylinder cavity using a rod parallel to the plates as shown in the figure. The cylinder and nozzle valves, which are pneumatically operated, are placed in plates 2 and 4, respectively. Because the piston assembly is retained in plate 3, the distance between plates 2 and 3 controls the volume. The part cavity is formed by plate 4 and plate 5. The ejection plates 7 and 8 are secured between plates 5 and 6, assisted by spacer block 6.

## Data Availability

Data are contained within the article.
